# Temporality of Features in Near-Death Experience Narratives

**DOI:** 10.3389/fnhum.2017.00311

**Published:** 2017-06-13

**Authors:** Charlotte Martial, Héléna Cassol, Georgios Antonopoulos, Thomas Charlier, Julien Heros, Anne-Françoise Donneau, Vanessa Charland-Verville, Steven Laureys

**Affiliations:** ^1^Coma Science Group, GIGA-Consciousness and Neurology Department, University of Liège and University Hospital of LiègeLiège, Belgium; ^2^Biostatistics, Public Health Department, University of Liège and University Hospital of LiègeLiège, Belgium

**Keywords:** near-death experience, narrative, temporality, feature, sequence, text analysis

## Abstract

**Background:** After an occurrence of a Near-Death Experience (NDE), Near-Death Experiencers (NDErs) usually report extremely rich and detailed narratives. Phenomenologically, a NDE can be described as a set of distinguishable features. Some authors have proposed regular patterns of NDEs, however, the actual temporality sequence of NDE core features remains a little explored area.

**Objectives:** The aim of the present study was to investigate the frequency distribution of these features (globally and according to the position of features in narratives) as well as the most frequently reported temporality sequences of features.

**Methods:** We collected 154 French freely expressed written NDE narratives (i.e., Greyson NDE scale total score ≥ 7/32). A text analysis was conducted on all narratives in order to infer temporal ordering and frequency distribution of NDE features.

**Results:** Our analyses highlighted the following most frequently reported sequence of consecutive NDE features: Out-of-Body Experience, Experiencing a tunnel, Seeing a bright light, Feeling of peace. Yet, this sequence was encountered in a very limited number of NDErs.

**Conclusion:** These findings may suggest that NDEs temporality sequences can vary across NDErs. Exploring associations and relationships among features encountered during NDEs may complete the rigorous definition and scientific comprehension of the phenomenon.

## Introduction

While the Near-Death Experience (NDE) phenomenon is still, at present, not fully understood, many ancient accounts and representations of these experiences date back to Plato’s Republic (Plato, 1937) and to the 15th century in Hieronymus Bosch’s paintings. At that time, these descriptions were, however, not designated as such. More recently, Moody (1975) popularized the phenomenon and its appellation through his best seller “*Life after life.*” Currently, NDEs are reported as a clearly identifiable physiological and psychological reality of clinical significance and can be referred to as “profound psychological events with transcendental and mystical elements typically occurring to individuals close to death or in situations of intense physical or emotional danger” (Greyson, 2000). Nowadays, an increased number of people claim to have had a NDE. Recent studies conducted among the general public of Australia (Perera et al., 2005) and Germany (Knoblauch et al., 2001) have estimated a prevalence of 4 to 8%. Moreover, it appears that 12 to 18% of cardiac arrest survivors have experienced NDEs –or at least some NDEs features (van Lommel et al., 2001; Greyson, 2003).

Near-Death Experiencers (i.e., people who have experienced a NDE; NDErs) usually report very detailed memories of their experiences (Thonnard et al., 2013; Palmieri et al., 2014) and, phenomenologically, NDEs can be described as a set of different and distinguishable features. In his book, Moody (1975) established a list of the 15 most frequently recounted features based on a recruited sample of 150 coma survivors. His 15-element model notably includes the overwhelming feeling of peacefulness and well-being, a sensation of being out of the body, the sight of a brilliant light and the feeling of being surrounded by it, life review, experiencing a tunnel, and decreased fear of death. His description of NDEs can actually be considered as the prevailing societal model of Western societies (Athappilly et al., 2006), notably because of the widespread popularity of his work. However, the author did not specify in his book any ranking of frequency or precise statistical data or figures. Recently, Charland-Verville et al. (2014) investigated NDEs features by using statistics and frequency distribution on reported responses to the Greyson NDE scale (Greyson, 1983) by retrospectively interviewing NDErs. They reported a ranked organization of the Greyson NDE scale features according to their frequency of occurrence: feeling of peacefulness/well-being, Out-of-Body Experience (OBE), seeing a bright light, altered time perception and entering unearthly environment. This frequency distribution is consistent with previous work: the feeling of peacefulness/well-being does indeed appear to be the most reported feature experienced during NDEs, followed by the OBEs (e.g., Greyson, 1990, 2003; Zhi-ying and Jian-xun, 1992; Pacciolla, 1996; Schwaninger et al., 2002; Lai et al., 2007; Corazza and Schifano, 2010). Conversely, precognitive visions seem to be the least recounted feature (e.g., Greyson, 1990, 2003; Zhi-ying and Jian-xun, 1992; Charland-Verville et al., 2014). Moreover, it seems that almost all NDE features tend to be more frequently reported by NDErs in retrospective studies –as compared to prospective ones (Charland-Verville et al., 2014).

The first documented attempt to establish a chronological order of NDE features was made through the observations of Ring (1980). He highlighted the recurrent NDE features of a sample of 102 individuals with a self-reported NDE and subsequently constructed the Weighted Core Experience Index (WCEI). The WCEI consists of a 10-point interview scale that allows researchers to differentiate between near-death and other experiences (by quantifying the depth of a NDE according to 10 arbitrarily weighted items and suggesting a cut-off point of 6 for identifying NDErs). Based on his scale, he noted that 48% of this sample had experienced a NDE. Derived from both this sample and his scale, Ring (1980) went one step further by proposing a 5-stages temporality sequence of NDEs: (1) “An experience of peace, well-being, and an absence of pain,” (2) “a sense of detachment from the physical body, progressing to an OBE,” (3) “entering darkness, a tunnel experience with panoramic memory, and a predominantly positive effect,” (4) “an experience of light that is bright, warm, and attractive,” and (5) “entering the light; meeting persons or figures.” He further suggested the concept of “non-core experiences” which are other types of features less frequently encountered during NDEs (e.g., encountering a presence or loved ones or life review). Although Ring is considered as pioneer with his work, his scale and the proposed 5-stages sequence have some limitations. In particular, the cut-off points were not based on statistical analysis and were not tested for internal coherence or reliability. Around the same period, Noyes et al. (1977) described 3 –instead of 5– successive phases: (1) resistance (including a recognition of danger, the fear of dying, a struggle to live, and acceptance of death), (2) life review, and (3) transcendence (i.e., a mystical state of consciousness). Some authors have thus decreased the ambiguity in descriptions of the phenomenological features and their frequency distribution. However, to date, no temporal schema has yet been rigorously identified.

The phenomenon of NDEs has been approached through diverse theoretical frameworks (mainly spiritual, psychological, or organic hypotheses; French, 2005) and some theories may now, at least in part, contribute to the explanation of specific NDE features –such as OBE or seeing a bright light. Neurobiological theories have notably proposed the potential implications of REM-sleep intrusions (Nelson et al., 2006), pharmacological factors (Jansen, 1989), altered blood gas levels (Klemenc-Ketis et al., 2010) as well as paroxistic temporal lobe disorders (Blanke et al., 2004; Britton and Bootzin, 2004; Hoepner et al., 2013). While sustained efforts have been made to better understand certain phenomenological features encountered during NDEs, the scientific literature devoted to the investigation of temporal structure of NDEs narratives seems rather limited. To the best of our knowledge, no study has formally and rigorously investigated whether NDE features follow a fixed order or distribution. Overall, the notion of temporality is fundamental to human experience. Indeed, temporality is central to characterizing narratives, because they are regularly developed in a dynamic temporal order when written (Fleischman, 1990) and their coherence emerges from this order (Trabasso et al., 1995). In the case of NDEs phenomenon, it can be noted that the temporal structure of narratives is dictated by each feature encountered by NDErs during the experience and configures those diverse features into a meaningful whole for the NDEr (Ring, 1980). The objective of the present study was to explore the chronology of NDE features in a sample of self-reported written narratives. In our view, investigating the temporality of NDE features may permit to highlight relationships and connections among them and, more generally, address the challenging question as to whether the patterns of NDEs are regular. Given a set of NDE narratives, the present study aimed at (1) exploring the frequency distribution of NDE features (overall frequency distribution of NDE features appearing in narratives, frequency distribution of the first and the last NDE feature occurrences, and frequency distribution of NDE features according to their position in the narratives); and (2) eventually identifying the most frequent stages of temporality sequences.

## Materials and Methods

### Participants and Procedure

Participants were recruited via the International Association for Near-Death Studies (IANDS France) and the Coma Science Group (GIGA-Consciousness, University of Liège and University Hospital of Liège, Belgium). Completion of the anonymous questionnaire was voluntary. Participants were then mailed a questionnaire that included items about socio-demographic (gender, age at NDE, age at interview) and clinical (time since NDE) characteristics. Participants were then asked to freely write down the detailed narrative of the experience on blank sheets of paper –without any text size restrictions. Finally, they were asked to respond to the Greyson NDE scale (Greyson, 1983). This scale is a validated (Greyson, 1983; Lange et al., 2004) 16-item multiple-choice tool used to quantify the intensity of the NDE (i.e., total score ranging from 0 to 32) and to permit a standardized identification of NDEs with a total score cut-off of 7. The Greyson NDE scale assesses core content components of 16 NDE features. For each item, the scores are arranged in an ordinal scale ranging from 0 to 2 (i.e., 0 = “not present,”1 = “mildly or ambiguously present,” and 2 = “definitively present”; Greyson, 1983; Lange et al., 2004). Participants whose experience did not meet the accepted criteria (i.e., total score < 7/32 on the Greyson NDE scale; Greyson, 1983) were therefore excluded from the present analysis. No incentive was offered for participation. This study was carried out in accordance with the recommendations of the ethics committee of the Faculty of Medicine of the University of Liège with written informed consent from all subjects. All subjects gave written informed consent in accordance with the Declaration of Helsinki. The protocol was approved by the ethics committee of the Faculty of Medicine of the University of Liège.

### Text Analysis

The first step consisted of the selection of the recurrent NDE features based on the literature and the experience of two experts gained by acquiring collecting and reading NDE testimonies. Before reading narratives, an initial list of the potential features described in literature (scientific publications and books) and reported to be characteristic of NDEs, was compiled. Firstly, the 16 key phenomenological features from the Greyson NDE scale (Greyson, 1983) were considered. Eight out of 16 Greyson NDE scale’s features were retained because each of them was clearly distinguished as one clear isolated feature (with a unique occurrence). The other remaining features were not retained to establish the chronology since they were considered by the research team as “diffuse” features. These words, describing the features, were spread throughout the narrative and a clear and precise position in the text was difficult to establish (e.g., altered time perception, extrasensory perception, heightened senses, unearthly environment). Then, the WCEI scale’s phenomenological features (Ring, 1980) were considered and two of them (not already reported in the Greyson NDE scale) were added in the list: Experiencing a tunnel and Entering the light. Lastly, the phenomenological feature Returning into the body was included as well for the text analysis, because it is generally reported by NDErs as a protruding element of NDEs and considered as a clear isolated feature. **Table 1** presents the final 11 NDE isolated features and the 5 other diffuse features retained to perform the text analysis.

**Table 1:** Classification of NDE features in written narratives.

	Description
**Isolated features**	
Out-of-Body-Experience	Experiencing a sense of detachment from the physical body, a perception of floating outside one’s body and/or perceiving one’s physical body from above –sometimes moving to other places.
Experiencing a tunnel	Entering, moving down or passing through a dark tunnel.
Feeling of peace	Experiencing a feeling a profound peace, well-being, calmness, pleasantness, happiness, joy, unconditional love, and/or an absence of pain.
Seeing a bright light	Seeing or feeling surrounded by a light that is white, bright, brilliant, warm and/or attractive –sometimes with a mystical and/or other-worldly origin.
Encountering with spirits/people	Meeting persons, being, presence figures and/or an voice (e.g., deceased loved ones, sacred figures, unrecognized beings, deceased or religious spirits, mystical beings) –sometimes with whom they communicate.
Life review	Reliving or watching some or the totality of their life history (e.g., past events, past actions).
Feeling of harmony	Experiencing a sense of harmony and/or unity with the nature and/or universe.
Coming to a border/point of no return	Approaching a border, a point of no return and/or a barrier –sometimes without access permissions.
Entering the light	Entering or going through the light.
Precognitive visions	Experiencing or watching scenes from personal and/or world’s future.
Returning into the body	Experiencing a decision by oneself or others to return to one’s body –often accompanied by feelings of reluctance.

**Diffuse features**	
Unearthly environment	Experiencing another, unearthly world and/or components of an another, unearthly world.
Heightened senses	Experiencing sensations more vivid than usual, sensations with all one’s senses or a crossover of senses.
Altered time perception	Experiencing a change in the perception of time (e.g., slowing down, speeding up, timelessness).
Extrasensory perception	Being aware of things or facts going on elsewhere –whether checked out (or not).
Speeded thoughts	Experiencing thoughts faster than usual.

After all written narratives were collected, the anonymous dataset was created. Some accounts constituted a couple of paragraphs and others spanned several pages. The average was about 383 words (ranged from 28 to 4411) per account. Text analysis was identically conducted on all written narratives. Two researchers (one expert and one novice unfamiliar with the NDE phenomenon) carefully and separately read all narrative texts from the dataset in order to understand subjective experiences and highlight each reported NDE feature. Without consulting each other, they broke narratives into constituent parts that all play integral roles in the narrative. This text analysis had two steps: (1) each feature explicitly stated in narrative texts (i.e., descriptive words or words sequences related to the feature) was isolated and classified into one of 16 categories (see **Table 1**). An open-vocabulary analysis (i.e., not requiring a predefined set of NDE keywords with a known correspondence to the NDE phenomenon) was conducted; (2) an order of appearance was then determined for each isolated feature –and not for the diffuse ones. All features were thus scored independently by the two experts. Finally, a dataset indicating whether the NDE (isolated or diffuse) feature was present or not –was used later to establish frequency distribution– and a stages temporality sequence for each narrative was acquired. After that, discrepancies among the analyzers (i.e., NDE features sequences without unanimity) were identified and then discussed between them until a consensus was reached.

### Statistical Analyses

#### Inter-rater Reliability

We used Cohen’s kappa coefficient to measure inter-rater reliability in order to assess the degree to which both researchers agree on their assessment decisions (the closer the value to 1, the better the concordance is between the two researchers).

#### Frequency Distribution of NDE Features

Frequency distribution was calculated from the dataset corresponding to narratives. Data analysis was carried out using SAS (version 9.3 for Windows) statistical package. We calculated overall frequency distribution of all NDE features appearing in narratives. By only using the isolated features, we also calculated frequency distribution of the first and the last NDE feature occurrences (i.e., the first and the last feature encountered by NDErs during the experience), and frequency distribution of NDE features according to their position in narratives.

#### Frequency Distribution of NDE Features Sequences

Frequency distribution of NDE features sequences was calculated from the dataset corresponding to narratives. Only isolated features were used for those analyses.

We first wanted to extract the most frequent sequences of two NDE consecutive features. For this, we used the four most frequent single features reported by NDErs –considering the threshold percentage of 50% (i.e., features reported in more than half of narratives)– and identified among them frequency distribution of each sequence of two consecutive features (that is, ultimately obtaining six pairs of two features).

We then wanted to extract the most frequent sequence consisting of four consecutive features –considering the obtained result that the mean number of NDE features per narrative was four (see Results section).

For these analyses, all temporality sequences were analyzed using a MatLab custom code which allowed us to observe the most frequent sequences of features reported in the narratives – among all sequences reported by NDErs and thus presented in the gathered dataset.

## Results

### Participants

We collected 154 French written narratives of NDEs (i.e., meeting the criteria: Greyson NDE scale total score ≥7/32; Greyson, 1983). The demographic data of the entire study cohort are presented in **Table 2**.

**Table 2:** Demographic data and Greyson NDE scale total score.

	Total *N* = 154
Gender–female	82 (53%)
Age at NDE (Mean in years ± SD)	34 ± 17
Age at interview (Mean in years ± SD)	55 ± 13
Time since NDE (Mean in years ± SD)	22 ± 15
Greyson NDE scale total score (Mean ± SD)	16 ± 6

### Inter-rater Reliability

Results showed an almost perfect agreement of both researchers for the text analysis with a Cohen’s kappa coefficient equal to 0.95 (95% confidence intervals 0.87–0.98).

### Frequency Distribution of NDE Features

Results were expressed as counts and proportions (%) for feature variables. This analysis showed that the mean number of NDE isolated features reported per narrative was 4 ± 2 (ranged from 1 to 9). The mean number of NDE diffuse features reported per narrative was 1 ± 1 (ranged from 0 to 4). When considering isolated and diffuse features, results revealed a mean number of 6 ± 2 (ranged from 1 to 15). The number and percentage of narratives in which each NDE feature appears –whatever their positions in the narrative text– are presented in **Table 3** (see the last column of the table). In all the narratives, the most frequently encountered NDE features were Feeling of peacefulness (80%) and Seeing a bright light (69%). The third most frequently reported NDE feature was Encountering with spirits/people (64%). The two least frequently reported NDE features were Speeded thoughts (5%) and Precognitive visions (4%).

**Table 3:** Frequency of the first and the last NDE isolated feature occurrences, and overall frequency of NDE (isolated and diffuse) features appearing in narratives (*N* = 154) –by decreasing order of frequency according to the first occurrence in narratives.

NDE features	First occurrence *N* (%)	Last occurrence *N* (%)	Overall frequency *N* (%)
**Isolated features**			
Out-of-Body-Experience	54 (35)	5 (3)	81 (53)
Experiencing a tunnel	36 (23)	3 (2)	73 (47)
Feeling of peace	24 (16)	22 (14)	123 (80)
Seeing a bright light	20 (13)	9 (6)	106 (69)
Encountering with spirits/people	8 (5)	17 (11)	99 (64)
Life review	7 (5)	8 (5)	25 (16)
Feeling of harmony	2 (1)	4 (3)	21 (14)
Coming to a border/point of no return	1 (1)	26 (17)	62 (40)
Entering the light	1 (1)	2 (1)	27 (18)
Precognitive visions	1 (1)	1 (1)	6 (4)
Returning into the body	0 (0)	56 (36)	57 (37)

**Diffuse features**			
Unearthly environment	–	–	57 (37)
Heightened senses	–	–	56 (36)
Altered time perception	–	–	54 (35)
Extrasensory perception	–	–	29 (19)
Speeded thoughts	–	–	8 (5)

**Table 3** also lists frequency distribution of the first and the last NDE feature encountered in written narratives. Results showed that the most frequent NDE feature appearing as the first feature in narrative texts was OBE (35%). The most frequent NDE feature appearing as the last feature –whatever the number of NDE features encountered during the experience– in narratives was Returning into the body (36%).

**Table 4** shows the frequency distribution of NDE features according to their position in the narratives. At time 1 (i.e., the first NDE feature appearing in narrative texts –whatever the total number of features encountered during the NDE), the most frequently reported feature was OBE (35%). At time 2(i.e., the second NDE feature appearing in narrative texts –whatever the total number of features encountered during the NDE), Feeling of peacefulness (31%) was the most often encountered feature. At time 3 and 4, the most frequently reported features were, respectively, Seeing a bright light (25%) and Encountering with spirits/people (24%). At time 5 and 6, the most frequently observed feature was Coming to a border/point of no return (respectively, 22 and 31%). At time 7, Returning into the body (56%) was the most often reported feature. At time 8, the two most frequently reported features were Coming to a border/point of no return and Returning into the body (both 37%). Finally, results demonstrated that only three narratives contain a ninth feature and all three were Returning into the body (100%). One can also observe in **Table 4** the total numbers of narratives contained at each occurrence time (see the last row of the table).

**Table 4:** Frequency of NDE features according to their position in narratives (total *N* represents the total number of narratives containing features in the corresponding occurrence time).

	Position in narratives									
NDE features	T1 *N* (%)	T2 *N* (%)	T3 *N* (%)	T4 *N* (%)	T5 *N* (%)	T6 *N* (%)	T7 *N* (%)	T8 *N* (%)	T9 *N* (%)
Feeling of peace	24 (16)	47 (31)^∗^	29 (22)	17 (17)	6 (9)	–	–	–	–
Seeing a bright light	20 (13)	35 (23)	33 (25)^∗^	15 (15)	2 (3)	–	–	1 (13)	–
Encountering with spirits/people	8 (5)	19 (13)	24 (18)	24 (24)^∗^	13 (19)	8 (20)	3 (12)	–	–
Out-of-Body-Experience	54 (35)^∗^	13 (9)	4 (3)	3 (3)	4 (6)	3 (8)	–	–	–
Experiencing a tunnel	36 (23)	21 (14)	11 (8)	3 (3)	1 (2)	1 (2)	–	–	–
Coming to a border/point of no return	1 (1)	5 (3)	14 (10)	9 (9)	15 (22)^∗^	12 (31)^∗^	3 (12)	3 (37)^∗^	–
Returning into the body	–	1 (1)	3 (2)	10 (10)	13 (19)	10 (26)	14 (56)^∗^	3 (37)^∗^	3 (100)^∗^
Entering the light	1 (1)	2 (1)	7 (5)	8 (8)	8 (12)	–	1 (4)	–	–
Life review	7 (4)	3 (2)	4 (3)	5 (5)	2 (3)	2 (5)	2 (8)	–	–
Feeling of harmony	2 (1)	5 (3)	2 (2)	4 (4)	3 (5)	3 (8)	1 (4)	1 (13)	–
Precognitive visions	1 (1)	–	2 (2)	2 (2)	–	–	1 (4)	–	–
Total	154 (100)	151 (100)	133 (100)	100 (100)	67 (100)	39 (100)	25 (100)	8 (100)	3 (100)

*^∗^The most frequent feature of the occurrence time.*

### Frequency Distribution of NDE Features Sequences

**FIGURE 1:**
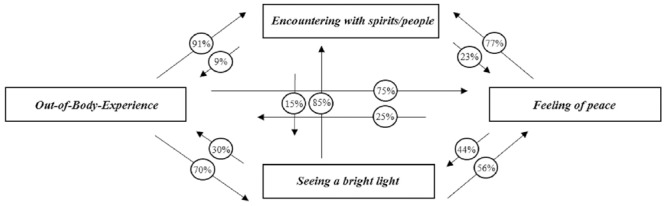
Frequency of 2 NDE features sequences in order of occurrence reported in narratives (values in parentheses are percentages calculated out of the total amount of narratives containing both NDE features).

Frequency distribution of each sequence of two consecutive features are presented in **Table 5** and **Figure 1**. Total values represent the total numbers of narratives containing both NDE features and frequency distribution percentages were calculated out of those totals. Both orders of occurrence for each pair of features are reported in **Table 5** and **Figure 1**.

**Table 5:** Frequency of 2 NDE features sequences in order of occurrence reported in narratives (percentages are calculated out of the total amount of narratives containing both NDE features).

NDE features sequences	Frequency *N* (%)
Out-of-Body-Experience → Encountering with spirits/people	43 (91)
Encountering with spirits/people → Out-of-Body-Experience	4 (9)
Total frequency	47 (100)

Seeing a bright light → Encountering with spirits/people	60 (85)
Encountering with spirits/people → Seeing a bright light	11 (15)
Total frequency	71 (100)

Feeling of peace → Encountering with spirits/people	63 (77)
Encountering with spirits/people → Feeling of peace	19 (23)
Total frequency	82 (100)

Out-of-Body-Experience → Feeling of peace	51 (75)
Feeling of peace → Out-of-Body-Experience	17 (25)
Total frequency	68 (100)

Out-of-Body-Experience → Seeing a bright light	37 (70)
Seeing a bright light →Out-of-Body-Experience	16 (30)
Total frequency	53 (100)

Seeing a bright light → Feeling of peace	49 (56)
Feeling of peace → Seeing a bright light	39 (44)
Total frequency	88 (100)

*Total frequency of narratives containing both NDE features.*

Our analysis demonstrated that, in 47 narratives containing both OBE and Encountering with spirits/people, 91% consecutively reported them in this order of occurrence. By contrast, only 9% reported both features in the opposite order. In 71 narratives containing both Seeing a bright light and Encountering with spirits/people, 85% encountered them in this order. Results also showed that 77% of narratives containing Feeling of peace and Encountering with spirits/people encountered both features in this order of occurrence. In 68 narratives containing both OBE and Feeling of peace, 75% reported them in this order. In all narratives containing both OBE and Seeing a bright light, 70% encountered both features in this order of occurrence and 30% reported them in the opposite order. The analysis finally showed that in all narrative texts containing both Seeing a bright light and Feeling of peace, 56% reported them in this consecutive order and 44% in the opposite order.

**FIGURE 2:**
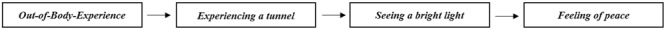
The most frequent sequence of four NDE features (22%) appearing in narratives (*N* = 27; percentage calculated out of the total amount of narratives containing these four NDE features).

Finally, **Figure 2** shows the most frequent sequence of four consecutive NDE features. Six (22%) out of the 27 narratives containing those four NDE features had this order of occurrence: OBE, followed by Experiencing a tunnel, followed by Seeing a bright light, ending by Feeling of peace. We find 33 other sequences of 4 consecutive features, but appearing in 2 to 5 narratives (the list being too exhaustive to be listed here).

## Discussion

The aim of this study was to examine frequency distribution of NDE features (frequency distribution of each single feature and according to their position in narrative texts) and NDE features sequences (i.e., the temporal order of distinct features) conducting text analysis on written narratives of self-reported NDEs (i.e., Greyson NDE scale total score ≥ 7/32; Greyson, 1983).

Firstly, our findings replicate previous research that has observed the feeling of peacefulness as the most frequently encountered feature during NDEs and precognitive visions as the less frequently encountered (Greyson, 1990, 2003; Zhi-ying and Jian-xun, 1992; Pacciolla, 1996; Schwaninger et al., 2002; Lai et al., 2007; Charland-Verville et al., 2014). Our results diverge, however, on the second most reported feature, which is Seeing a bright light in the present study. OBE is here recorded in 53% of the testimonies (i.e., the fourth more frequent feature) while it is usually reported in the literature as the second most commonly encountered feature in NDEs (i.e., about 80%; Greyson, 1990, 2003; Schwaninger et al., 2002; Lai et al., 2007; Corazza and Schifano, 2010; Charland-Verville et al., 2014). Moreover, we observe that OBE is the most frequently cited feature at the very beginning of the narratives and Returning into the body at the very end. This suggests that NDEs seem to be regularly triggered by a sense of detachment from the physical body and end when returning to one’s body. More generally, we observe that NDEs narratives vary in “richness” of encountered features, more specifically, some narratives may include one feature while (remarkably rich) others may include up to 15 features in a single experience. Ultimately, the most significant features (i.e., occurring > 50%) identified in the present work include Feeling of peace, Seeing a bright light, Encountering with spirits/people and OBE. Based on the present results –and consistent with previous literature (e.g., Greyson, 2003; Lai et al., 2007; Charland-Verville et al., 2014), it appears that no NDE feature is universal in its occurrence.

Our next goal was to investigate frequency distribution of consecutive NDE features. Our results show that the most frequently reported sequence of two consecutive features is, in order of appearance, Feeling of peace and Encountering with spirits/people. Interestingly, it also appears that Seeing a bright light, OBE and Feeling of peace are all the more regularly followed by Encountering with spirits/people in narratives (see **Figure 1**). We further observe that NDErs experience more often an OBE before experiencing a Feeling of peace –than the opposite pattern. Finally, in contrast to all other pairs of features, the order of occurrence of both features Seeing a bright light and Feeling of peace seems less clearly manifested (i.e., almost similar percentages observed in both orders) –although the overall occurrence frequency of this pair is higher than the other pairs. We then suggest that it could be due to a strong association between those both features. It has been previously suggested that the bright light spotted by NDErs is regularly associated with a profound feeling of peace (Moody, 1975; Corcoran, 1988; often described as “light peace” and extremely pleasant) and/or love (Facco and Agrillo, 2012). Therefore, this may make it difficult for NDErs to clearly distinguish both features and then identify a chronological order among them.

Third, the present results highlight the most frequent temporality core features sequence reported by NDErs in their narratives: OBE, followed by Experiencing a tunnel, followed by Seeing a bright light, finally followed by Feeling of peace. We find nevertheless this sequence in a relatively small number of accounts (i.e., 6). Actually, no invariable temporal sequence of features (i.e., observed in all or at least most narratives) could be established in our sample of narratives, suggesting that every NDEr might report a unique pattern of experience. We then could consider NDEs narratives as a changeable collection of possible elements differing according to NDErs –and not as a regular pattern. While NDEs may have a universal character so that they may exhibit enough common features to belong to the same phenomenon (e.g., Grosso, 1981; Atwater, 1988; Charland-Verville et al., 2014), we nevertheless observe in this study a temporal variability within the distribution of reported features. Indeed, our findings suggest that NDEs may not feature all elements and elements do not seem to appear in a fixed order. This raises significant questions about what specific aspects of NDEs could be considered as universal –and what not. Further research is necessary to explore differences across NDErs and the precise extent of which content of those experiences reflects their expectations and cultural backgrounds.

In parallel, the text analysis highlights “diffuse” features; that is, dimensions which are reported using words spread throughout narratives. We observe that the most frequently encountered diffuse feature is Unearthly environment and the less frequently one is Speeded thoughts. Moreover, our results reveal that diffuse features are, in general, less frequently reported than isolated features. The former are reported by NDErs with a frequency ranging from 5 to 37%, while the latter are observed in testimonies with a frequency ranging from 4 to 80%. Interestingly, the text analysis reveals the impossibility of establishing a clear and precise position for diffuse features because of the propagation of words through narratives. For some of them, this lack of localization may be surprising (e.g., Unearthly environment) and further studies should investigate both kind of features: features referring to isolated events and features characterizing the structure of the whole narrative.

Alternatively, we believe that our findings are significant by inferring the relative order of NDE features reported in narratives so as to observing existing associations and relationships among them in the whole experience. NDEs include distinct and specific yet unexplained cognitive experiences (physiologically real and considered as features in the present study), which may possibly underlie different cerebral mechanisms. Some authors have suggested that the mechanisms involved during NDEs led to a “cascade of events” resulting in the occurrence of diverse NDE features (Blanke and Dieguez, 2009). More generally, a better understanding of the existing relationships among NDE features (i.e., determine how they are interrelated) might help us to explain the entire phenomenon of NDEs and its underlying mechanisms. The temporality is fundamental because it concerns our reported perception of the time passing during experiences, which is one of the central aspects of consciousness (Arstila, 2012). Indeed, it refers to the perception of all the different experiences we have lived and how we will later recall them. Phenomenologically, life-threatening situations are commonly associated with alteration in the experience of time and space (Tart, 1972; Baruss, 2003; Arstila, 2012) as well as bodily perception (Tart, 1972; Ataria and Neria, 2013). Several (non-exclusive) assumptions suggest that as a result of stress, our senses could record stimuli at higher density, our brain could process more quickly stimuli and/or our memory could store stimuli at higher density (Stetson et al., 2007; Arstila, 2012). By contrast, it has also been postulated that “time appears to slow down because richer than usual memories are later erroneously interpreted to have spanned a greater period of time than the experience on which they are based actually did” (Arstila, 2012). Because notions of time and chronology are constructs closely related to memory and consciousness, the present observation that NDE features appear in a variable order may be relevant in the refinement of their definition. NDEs are complex experiences and, in our opinion, it is also essential to consider the experience as a whole (i.e., explore and capture all its components and how they are interrelated) for a better comprehension of the phenomenon. In this paper, we therefore offer a first look at the temporal dimension of features in NDEs accounts. We observe in this study a possible coexistence of different ways to describe features and their temporality (i.e., isolated versus diffuse features) within narrations. Further investigations are needed to examine the neurophysiological mechanisms underlying both types of features. It would also be interesting to observe whether the sequences of features highlighted in the narratives accurately reflect their perceived order of appearance during the experience and/or are the result of a (*a posteriori*) temporal way of describing a timeless –or at least, time-distorted– experience. More generally, investigating both the phenomenology and the situations where such time distortions occur continues to represent a methodological challenge and we think that investigators must explore and rely on testimonies in a quest to reveal patterns relevant to develop theories.

There are some limitations in this study that deserve mentioning. First, limitation is the extent to which testimonies we received are skewed by a selection bias. Indeed, these findings might not reflect the absolute frequency since many NDErs can be uncomfortable sharing their experience. Nevertheless, our study includes a relatively large sample of testimonies. Second, we still do not know exactly to what extent the accounts we got are influenced by the models and the representations of the phenomenon through the media and published work [e.g., NDE description in Moody’s (1975) book]. In general, the question of the reliability of NDEs accounts still remains relatively unexplored. Furthermore, it should be stressed that our collection of narratives were written in the same language (in this case, French). It would be very interesting to compare NDE narratives from different languages in order to better investigate the challenging question of socio-cultural influence. Finally, although retrospectively reported NDE narratives seem to have a different content compared to the prospectively reported ones (Charland-Verville et al., 2014), it could be interesting to investigate the temporality of features encountered in retrospective versus prospective studies.

## Conclusion

The present study highlights the recurrent sequences of NDE features reported in narratives and shows that NDEs’ features do not appear in a strict temporal order, but rather in a variable (i.e., differ across NDErs) one. In our opinion, the presented data emphasizes and grants the uniqueness of NDErs’ experiences. We think that NDEs, as a complex set of phenomena, remain of considerable interest to neurosciences for the current understanding of consciousness.

## Authors Contributions

CM, HC, and VC-V have followed data acquisitions. CM, GA, TC, JH, and A-FD have analyzed the data. CM, HC, VC-V, and SL have interpreted results. CM, VC-V, and SL have drafted the manuscript and designed the study. All authors have critically revised the manuscript and approved the final version to be published and agreement to be accountable for all aspects of the work.

## Conflict of Interest Statement

The authors declare that the research was conducted in the absence of any commercial or financial relationships that could be construed as a potential conflict of interest.
