# Corrigendum: Temporality of Features in Near-Death Experience Narratives

**DOI:** 10.3389/fnhum.2017.00435

**Published:** 2017-08-22

**Authors:** Charlotte Martial, Héléna Cassol, Georgios Antonopoulos, Thomas Charlier, Julien Heros, Anne-Françoise Donneau, Vanessa Charland-Verville, Steven Laureys

**Affiliations:** ^1^Coma Science Group, GIGA-Consciousness and Neurology Department, University of Liège and University Hospital of Liège Liège, Belgium; ^2^Biostatistics, Public Health Department, University of Liège and University Hospital of Liège Liège, Belgium

**Keywords:** near-death experience, narrative, temporality, feature, sequence, text analysis

In the original article, the name of one of the editors was incorrectly spelled in the reference for “Greyson B. (2000). “Near-death experiences,” in *Varieties of Anomalous Experience: Examining the Scientific Evidence*, eds E. Cardefia, S. J. Lynn, and S. Krippner (Washington, DC: American Psychological Association).” as “Cardefia”. It should be “Cardeña.” The authors apologize for this error and state that this does not change the scientific conclusions of the article in any way.

## Conflict of interest statement

The authors declare that the research was conducted in the absence of any commercial or financial relationships that could be construed as a potential conflict of interest.
